# Genotoxic potential of diesel exhaust particles from the combustion of first- and second-generation biodiesel fuels—the FuelHealth project

**DOI:** 10.1007/s11356-017-9995-0

**Published:** 2017-09-09

**Authors:** Magdalena Kowalska, Aneta Wegierek-Ciuk, Kamil Brzoska, Maria Wojewodzka, Sylwia Meczynska-Wielgosz, Joanna Gromadzka-Ostrowska, Remigiusz Mruk, Johan Øvrevik, Marcin Kruszewski, Anna Lankoff

**Affiliations:** 1 Department of Radiobiology and Immunology, Institute of Biology, Jan Kochanowski University, 15 Swietokrzyska Str, 25-406 Kielce, Poland; 2 Center for Radiobiology and Biological Dosimetry, Institute of Nuclear Chemistry and Technology, 16 Dorodna Str, 03-195 Warsaw, Poland; 3 Faculty of Human Nutrition and Consumer Science, Warsaw University of Life Sciences, 166 Nowoursynowska Str, 02-787 Warsaw, Poland; 4 Faculty of Production Engineering, Warsaw University of Life Sciences, 166 Nowoursynowska Str, 02-787 Warsaw, Poland; 5 Domain of Infection Control and Environmental Health, Norwegian Institute of Public Health, P.O. Box 4404, Nydalen, 0403 Oslo, Norway; 6 Department of Molecular Biology and Translational Research, Institute of Rural Health, Jaczewskiego 2, 20-090 Lublin, Poland; 7 Faculty of Medicine, University of Information Technology and Management in Rzeszow, Sucharskiego 2, 35-225 Rzeszow, Poland

**Keywords:** Diesel exhaust particles, First- and second-generation biodiesel fuels, Single- and double-strand breaks, Oxidative DNA damage, Chromosomal damage

## Abstract

Epidemiological data indicate that exposure to diesel exhaust particles (DEPs) from traffic emissions is associated with higher risk of morbidity and mortality related to cardiovascular and pulmonary diseases, accelerated progression of atherosclerotic plaques, and possible lung cancer. While the impact of DEPs from combustion of fossil diesel fuel on human health has been extensively studied, current knowledge of DEPs from combustion of biofuels provides limited and inconsistent information about its mutagenicity and genotoxicity, as well as possible adverse health risks. The objective of the present work was to compare the genotoxicity of DEPs from combustion of two first-generation fuels, 7% fatty acid methyl esters (FAME) (B7) and 20% FAME (B20), and a second-generation 20% FAME/hydrotreated vegetable oil (SHB: synthetic hydrocarbon biofuel) fuel. Our results revealed that particulate engine emissions from each type of biodiesel fuel induced genotoxic effects in BEAS-2B and A549 cells, manifested as the increased levels of single-strand breaks, the increased frequencies of micronuclei, or the deregulated expression of genes involved in DNA damage signaling pathways. We also found that none of the tested DEPs showed the induction of oxidative DNA damage and the gamma-H2AX-detectable double-strand breaks. The most pronounced differences concerning the tested particles were observed for the induction of single-strand breaks, with the greatest genotoxicity being associated with the B7-derived DEPs. The differences in other effects between DEPs from the different biodiesel blend percentage and biodiesel feedstock were also observed, but the magnitude of these variations was limited.

**Electronic supplementary material:**

The online version of this article (10.1007/s11356-017-9995-0) contains supplementary material, which is available to authorized users.

## Introduction

The overall impact of engine emissions on human health has been studied for a long time, mostly due to the presence of polycyclic aromatic hydrocarbons (PAHs) and their derivatives (nitro-PAHs) in diesel exhaust particles (DEPs). Epidemiological data indicate that exposure to DEPs from traffic emissions is associated with a higher risk of morbidity and mortality related to cardiovascular and pulmonary diseases (Vieira et al. 2017; Steiner et al. 2016). There is also compelling evidence from animal experimental models and humans that exposure to DEPs and ambient air particles is associated with accelerated progression of atherosclerotic plaques (Cao et al. 2016). Recently, evidence has also been obtained about their possible carcinogenicity (Hesterberg et al. 2012). While the impact of DEPs from combustion of fossil diesel fuel on human health has been extensively studied, current knowledge of DEPs from combustion of biodiesels provides limited and inconsistent information about their mutagenicity and genotoxicity, as well as possible adverse health risks. The vast majority of studies comparing DEPs from combustion of fossil diesels and biodiesels used the bacterial reverse mutation assay (so-called Ames test). Early research on mutagenicity of DEPs in *Salmonella typhimurium* tester strains TA98 (frame-shift mutation) and TA100 (base-pair substitution) has shown that biodiesel emissions induced subtly lower mutagenic potency when compared to the exhaust of fossil fuels (Bünger et al. 1998; Westerholm et al. 2001). Other studies revealed either no differences or significant increase in mutagenic activity of biodiesel emission extracts (Westphal et al. 2012; Krahl et al. 2008). The more recent studies revealed a clear reduction of mutagenic effects, showing less differences between the biodiesels compared to reference fuels (Bünger et al. 2012). It is believed that the main contributors to the mutagenicity of biodiesel DEPs are the PAH and nitro-PAH compounds adsorbed onto the particle surface. Besides the mutagenicity determined in a prokaryote model, genotoxic properties of biodiesel-derived DEPs have also been evaluated in eukaryote models. Two studies dealing with the formation of bulky DNA adducts have shown contradictory results. Ross et al. (2015) reported the formation of multiple DNA adducts by the in vitro metabolic activation of organic extracts of DEPs from combustion of fossil diesel and soy biodiesel. However, no formation of DNA adducts was presented in vitro by André et al. (2015), even if cells were fully able to metabolize nitroaromatics and PAHs. Other in vitro studies revealed the induction of DNA strand breaks, but either no differences or significant differences were observed between DEPs from combustion of fossil diesel and biodiesel (Jalava et al. 2010; Hemmingsen et al. 2011; Jalava et al. 2012). Similarly, inconsistent results were observed for the induction of micronuclei by DEPs from combustion of fossil diesel and biodiesel, showing either no difference or significant increase of chromosomal DNA damage by biodiesel-derived DEPs (Leme et al. 2012; Cervena et al. 2016). In summary, these contradictory trends are hard to interpret and may be caused by chance in light of relatively limited data. Moreover, a comparison of the results of different toxicological studies for exhaust particles produced by biodiesel combustion is difficult because of differences in the experimental approach, including age and type of diesel engine, drive cycle, feedstock blend, and its percentage in the blended fuel. The objective of the present work was to compare the genotoxicity of different DEPs from combustion of first- and second-generation biodiesel fuels in relation to their physicochemical properties. DEPs were produced by the 1.3 JTD engine (Euro V stage), fueled with three biodiesel fuels of commercial interest: the first-generation B7 biodiesel fuel (7% fatty acid methyl esters (FAME)), which is currently used in the EU; the first-generation B20 biodiesel fuel (20% FAME); and the second-generation (synthetic hydrocarbon biofuel (SHB)) biodiesel fuel (7% FAME and 13% synthetic hydrotreated vegetable oil (HVO)). These biodiesel fuels were combusted under identical engine operation conditions, and emissions were evaluated during a certified test cycle. Detailed physicochemical characterizations of DEPs were performed to investigate how the composition of three types of DEPs affects their biological effects in vitro, measured as induction of single- and double-strand breaks, oxidative DNA damage, and chromosomal damage. In addition, the expression of genes involved in DNA damage signaling was also evaluated to screen for possible molecular mechanisms of toxicity.

## Materials and methods

### Collection of DEPs

A Fiat Panda with compression ignition engine 1.3 JTD (common rail third-generation injection system, engine capacity 1248 cm^3^, max power 75 bhp, max torque 190 Nm, production year 2014), fulfilling the requirements of the Euro V stage, was used as a DEP source. The engine was tested under controlled conditions on a chassis dynamometer (Schenck Komeg EMDY 48) at constant engine speed and load of 340 rpm and 45.7%, respectively, corresponding to a constant vehicle speed of 43.75 km/h. The engine temperature was kept at 94 °C during the test cycle. The engine was fueled by three different mixtures of diesel oil and biocomponents: (1) first-generation biodiesel fuel “B7,” containing 7% vol. FAME in diesel oil; (2) first-generation biodiesel fuel “B20,” containing 20% vol. FAME in diesel oil; and (3) second-generation biodiesel fuel “SHB,” containing 13% vol. synthetic HVO (NExBTL) and 7% vol. FAME in diesel oil. According to the newest EU Directive 2015/1513 that encourages the use of second-generation biofuels (such as HVO), instead of first-generation biofuels (such as FAME and Rapeseed oil methyl ester (RME)), we chosen these two types of biofuels: HVO which is Neste Renewable biodiesel (formerly NEXBTL), produced in a patented vegetable oil refining process and commercialized by the Finnish oil and refining company Neste (Rantanen et al. 2005) and FAME, which is currently used in EU and produced in the process of transesterification of plant oils with methanol by ORLEN Poland. The DEPs used in the present study were collected from the main diesel exhaust without a diesel particle filter on PTFE-coated glass fiber filters (Pallflex, Emfab filters, TX40HI20WW, 70 mm). For the quantitative analysis, the blank sampling filters were marked and weighed. The filters from individual fuels were separately re-weighed after sampling, and the mass differences before and after the sampling were compared and analyzed to determine the total particulate matter collected. Driving cycles and particulate collections were repeated with the same fuel several times to new filter sets to ensure that a sufficiently large sample mass would be obtained in order to complete the toxicological and chemical analyses. Filters were stored in the freezer at − 20 °C for 1 week before analysis*.* Particles for in vitro experiments were scraped from the filters using a clean stainless steel blade.

### Preparation of diesel exhaust particles

DEP-stock solutions were prepared by dispersion of 2 mg of particles in 1 mL of LHC-9 serum-free medium (for experiments with BEAS-2B cells) or in 1 mL of F12 Ham medium supplemented with 10% FCS (for experiments with A549 cells). DEP-dispersions were then sonicated on ice using the Sonic Vibra Cell ultrasonic liquid processor (USA). Ultrasonic energy (3 kJ) was provided in pulses (30 s on, 10 s off) at 60% amplitude. Stock solutions were dispensed (100 μL) into sterile 1-mL cryogenic vials and stored at – 20 °C. The samples were thawed before each set of experiments at 37 °C for 60 s, dispersed in the corresponding medium at a ratio of 1:10, and mixed prior to use (working solution).

### Physicochemical characterization of diesel exhaust particles

Physicochemical characterization of diesel exhaust particles was described in detail by Lankoff et al. (2017). Briefly, sample size distribution was measured by the Nanoparticle Tracking Analysis (NTA) with a NanoSight LM20 (NanoSight, Amesbury, UK), equipped with a sample chamber with a 640-nm laser. Zeta potential and polydispersity index were determined by DLS method at 25 °C in a folded capillary cell at 150 V and M3-PALS detection using non-invasive backscatter at 173° with an Avalanche photodiode, Q.E. > 50% at 633 nm (Malvern, Malvern Hills, UK). The shape of DEPs was analyzed by transmission electron microscopy (TEM) (JEOL 1200 EXII, JEOL, JAPAN) operating at an acceleration voltage of 120 kV. Elemental analysis of DEPs was performed by digital scanning electron microscopy (SEM) type DSM 942 (Zeiss, Germany) in the secondary electron (SE) mode using the energy dispersive X-ray spectrometry (EDS) with Quantax 400 (Bruker, Germany) system. Separation and analysis of PAHs from particulate extracts were described in detail by Czarnocka and Odziemkowska (2016). The content of 17 PAHs was measured by the Agilent 7890A GC System chromatograph coupled with a mass spectrometer MS 5975C using a low-polarity Rtx-5ms capillary column (30 m × 0.25 mm × 0.25 μm) (Restek, Bellefonte, PA, USA).

### Cell cultures

The human type-II-like alveolar epithelial cell line A549 and the human bronchial epithelial cell line BEAS-2B were purchased from the American Type Tissue Culture Collection (ATCC, Rockville, MD) and maintained according to ATCC protocols. Briefly, A549 were cultured in F12 Ham medium supplemented with 10% FCS and 2 mM L-glutamine, whereas BEAS-2B were cultured in LHC-9 serum-free bronchial epithelial growth medium on non-coated plates. Both cell lines were maintained in an incubator at 37 °C with 5% CO_2_. The exponentially growing BEAS-2B and A549 cells were incubated with 1, 10, 25, and 50 μg/mL of DEPs for 6, 24, or 48 h (depending on the assay procedure). The doses and treatment times were chosen based on the cytotoxicity results (e.g., induction of apoptosis and necrosis, inhibition of protein synthesis, generation of free radicals), published previously by our group (Lankoff et al. 2017).

### Determination of single-strand DNA breaks and oxidative DNA damage by the comet assay

The exponentially growing BEAS-2B and A549 cells were incubated with 1, 10, 25, and 50 μg/mL of DEPs for 24 h. Positive control cells were irradiated with a dose of 2 Gy of X-rays at a dose rate of 1.14 Gy/min (Xylon International Smart 200-E irradiator, Xylon, San Jose, CA). The comet assay (single cell gel electrophoresis) was performed as previously described (Wojewódzka et al. 1998). Briefly, an aliquot of cell suspension was mixed with an equal volume of 2% low melting point agarose (type VII, Sigma), put on a microscope slide pre-coated with 0.5% regular agarose (type I-A, Sigma), and left on ice. After agarose solidification, the slides were immersed in a cold lysing solution (2.5 M NaCl, 100 mM Na_2_EDTA, 10 mM Tris, and 1% Triton X-100, pH 10) or left for 30 min in culture media at 37 °C to allow damage repair. After 40 min lysis, the slides were placed on a horizontal gel electrophoresis unit filled with fresh electrophoretic buffer (1 mM Na2EDTA (sodium ethylenediamine tetraacetate) and 300 mM NaOH) and allowed to stay in this buffer for 40 min for DNA unwinding. Next, electrophoresis was performed (1.2 V/cm, 30 min, 10 °C). After electrophoresis, the slides were washed with 0.4 M Tris, pH 7.5 (3 × 5 min), and stained with DAPI (4′,6-diamidino-2-fenylindole), 50 μL (1 μg/mL). Basically, the same test was applied for the measurement of DNA base damage. Incubation of irradiated cells with the formamidopyrimidine glycosylase (FPG), BioLabs, was carried out as previously described (Kruszewski et al. 1998). Briefly, after lysis, the slides were washed 3 × 5 min with the buffer (40 mM Hepes (4-(2-hydroxyethyl)-1-piperazineethanesulfonic acid), 0.1 M KCl, 0.5 mM EDTA, 0.2 mg/mL bovine serum albumin, pH 8) at 4 °C. Further, 50 μL of FPG solution (4.8 × 10–2 U) in the buffer was placed on each slide, covered with cover glass, and incubated for 30 min in a light-protected box at 37 °C. Slides were stained with DAPI (1 μg/mL) and analyzed as described above. Image analysis of data was performed by the Comet Assay IV image analysis system (Perceptive Instruments, UK). Data for 75 randomly selected comets per point were analyzed. Percent of DNA in comet’s tail was chosen as a measure of DNA damage.

### Analysis of double-strand DNA breaks by the γ-H2AX assay

BEAS-2B and A549 cells at exponential growth were incubated with 1, 10, 25, 50, and 100 μg/mL of the different DEPs for 24 h. Positive control cells were irradiated with a dose of 2 Gy of X-rays at a dose rate of 1.14 Gy/min (Xylon International Smart 200-E irradiator, Xylon, San Jose, CA). γ-H2AX foci were detected with the γ-H2AX (H2A.X PHOS) Detection Kit (Upstate Biotechnology, USA). Briefly, after incubation, the cells were washed, fixed, and resuspended in a permeabilization solution (0.5% saponin, 10 mM HEPES, 140 mM NaCl, 2.5 mM CaCl2). Unspecific binding was blocked for 1 h in blocking buffer BSA-T-PBS (1% BSA, 0.1% Triton X-100 in PBS). Thereafter, the cells were suspended in BSA-T-PBS containing 2 μg of the fluorescein-conjugated γ-H2AX antibody (monoclonal anti-phosphohistone H2AX antibody, Upstate Biotechnology) for 20 min. Cells were analyzed with a FACScan (Becton Dickinson, San Jose, CA, USA). Twenty thousand cells per point were analyzed for γ-H2AX intensity.

### Determination of chromosomal DNA damage by the micronucleus assay

The exponentially growing BEAS-2B and A549 cells were incubated with 1, 10, 25, and 50 μg/mL of DEPs for 24 and 48 h. Positive control cells were irradiated with a dose of 2 Gy of X-rays at a dose rate of 1.14 Gy/min (Xylon International Smart 200-E irradiator, Xylon, San Jose, CA). After 1 h of treatment, cytochalasin-B (final concentration of 10 μg/mL) was added into the cell culture medium in order to block cytokinesis and obtain binucleated cells. Cytochalasin-B is an inhibitor of microfilament ring assembly required for the completion of cytokinesis. This inhibition allows to generate once-divided binucleated cells, which are the cells that can express micronuclei. Restricting scoring of micronuclei in binucleated cells prevents confounding effects caused by suboptimal or altered cell division kinetics, which is a major variable in the micronucleus assay protocol that does not distinguish between non-dividing cells that cannot express micronuclei and dividing cells that can (Fenech 2007). After the exposure, the cells were harvested by centrifugation and subjected to cold mild hypotonic treatment (0.075 M KCl) for 8 min, fixed twice with methanol/acetic acid/ringer solution (13:12:3), and then dropped on clean, dry slides. The cells were mounted and stained in the Vectashield Mounting Medium containing DAPI (4′,6-diamidyno-2-fenyloindol). The slides were coded, and the frequency of micronuclei (MN) in 3000 binucleated cells (BNC) per dose (1000 cells/replicate) was analyzed with the fully automated image acquisition and analysis system Metafer (Metasystems, Germany) by one skilled scorer. The frequency of MN in untreated controls and positive controls (2 Gy) was also scored in 3000 BNC (1000 cells/replicate), according to the criteria proposed by Fenech (2007).

### RNA isolation, reverse transcription, and real-time PCR

Total RNA was extracted from cell pellets using the RNeasy Mini Kit (Qiagen) according to manufacturer’s protocol. To assess the concentration and purity of RNA, the portion of every RNA sample was diluted in TE buffer (pH 8.0) and the absorbance at 230, 260, and 280 nm was measured using Cary 50 UV-Vis spectrophotometer (Varian). All RNA samples used in subsequent analyses had a concentration ≥ 100 ng/μL, as well as A260/A280 and A260/A230 ratios ≥ 2.0. RNA integrity was tested by agarose gel electrophoresis. For PCR array analysis, 1 μg of total RNA was converted to complementary DNA (cDNA) in a 20-μL reaction volume using RT^2^ First Strand Kit (Qiagen). The cDNA was diluted with 91 μl distilled water and used for the expression profiling using the human DNA damage signaling pathway PCR array (Qiagen, cat. no. PAHS-029Z) according to manufacturer’s instructions. Briefly, a total volume of 25 μL of PCR reaction mixture, which included 12.5 μL of RT^2^ SYBR Green/ROX qPCR Master Mix from Qiagen (containing HotStart DNA Taq polymerase, SYBR Green dye and the ROX reference dye), 11.5 μL of double-distilled H_2_O, and 1 μL of diluted template cDNA, was used for each primer set in each well of the PCR array. One technical replicate was performed for each sample. PCR amplification was carried out using 7500 Real-Time PCR System (ThermoFisher Scientific) with an initial 10-min step at 95 °C followed by 40 cycles of 95 °C for 15 s and 60 °C for 1 min. Relative gene expression was calculated using the ΔΔCt method with ACTB, B2M, GAPDH, HPRT1, and RPLP0 as reference controls. Calculations were done using the Relative Quantification Software version 3.2.1-PRC-build1 (Thermo Fisher Cloud). Statistical differences were examined by Student’s *t* test with *p* < 0.05 considered to be statistically significant.

### Statistical evaluation

Statistical analysis of the obtained data was performed using the Statistica 7.1 software (Stat Soft. Inc., Tulsa, USA). The data were expressed as mean ± standard deviation (SD) of at least three independent experiments. Data were evaluated by Kruskal-Wallis one way analysis of variance on ranks (ANOVA) followed by the post hoc Fisher’s test. Correlation coefficients between the obtained data were evaluated by the Pearson product-moment. Differences were considered statistically significant when the *p* value was less than < 0.05.

## Results

### Physicochemical characterization of DEPs

A detailed physicochemical characterization of the DEPs used in this study has previously been published (Lankoff et al. 2017. As shown in Table 1, the average hydrodynamic diameters of all three types of DEPs were comparable, if the same culture medium was used. However, the B7 biofuel generated the lowest number of particles with diameter in the range 1–90 nm (~ 55%), as compared with the B20 biofuel (~ 70%) and the SHB biofuel (~ 85%). The polydispersity index values for all DEPs were less than 0.5, indicating high homogeneity of the suspension. The zeta potentials were negative and comparable for all tested DEPs, indicating stability of the colloidal system. TEM analysis revealed that all three types of DEPs were nearly spherical, and the particles were present as single particles, clusters, or chain-like aggregates. All DEPs were composed primarily of carbon (~ 85%). The next most abundant elements were oxygen and nitrogen. Zinc was present in all DEPs at low concentration. Concentrations of sulfur, copper, and chlorine were below 0.5% in all samples, with the exception of chlorine in SHB-DEP, which was slightly above 1%. Silver and iron could only be detected in SHB-DEP samples. The content of PAHs in DEP-derived organic extracts was the highest in the B7-DEP sample (165.78 ng/mg) and the lowest in the SHB-DEPs sample (69.93 ng/mg). The data showed that pyrene, fluoranthene, phenanthrene, and chrysene were the most abundant PAHs in all samples.

**Table 1:** Physicochemical characteristics of diesel exhaust particles from the combustion of B7, B20, and SHB biodiesel fuels. Elemental composition of DEPs (wt%), PAHs (ng/mg) in the organic extracts from DEPs, particle size (nm), polydispersity index, and zeta potential (mV) of B7-DEPs, B20-DEPs, and SHB-DEPs in cell culture medium F12+FBS or LHC-9. All data presented as mean of triplicates ± SD

Chemical constituents of the particulate samples	Diesel exhaust particles		
	B7-DEP	B20-DEP	SHB-DEP
Elemental composition (wt%)			
Carbon Oxygen Nitrogen Zinc Sulfur Copper Chlorine Silver Iron	85.53 ± 0.93 7.51 ± 1.02 5.12 ± 0.97 1.40 ± 0.44 0.22 ± 0.04 0.18 ± 0.03 0.04 ± 0.01 – –	86.76 ± 1.09 6.31 ± 1.21 5.15 ± 0.87 1.24 ± 0.05 0.22 ± 0.04 0.19 ± 0.08 0.10 ± 0.02 – –	87.51 ± 0.86 2.24 ± 0.85 5.11 ± 0.85 2.25 ± 0.77 0.26 ± 0.07 0.45 ± 0.21 1.33 ± 0.36 0.72 ± 0.12 0.21 ± 0.03
Organic components (ng/mg)			
Naphthalene Acenaphthylene Acenaphthalene Fluorine Phenanthrene Anthracene Fluoranthene Pyrene Benzo(a)anthracene Chrysene Benzo(b)fluoranthene Benzo(k)fluoranthene Benzo(a)pyrene Benzo(a)fluoranthene Indeno(1,2,3-c,d)pyrene Dibenzo(a,h)anthracene Dibenzo(g,h,i)perylene Total PAHs	3.86 0.26 0.11 0.56 12.33 1.13 36.04 90.33 2.07 10.26 4.30 2.59 0.38 0.65 0.36 0.07 0.47 165.78	7.74 0.92 0.19 1.41 20.83 1.73 6.06 43.23 1.14 3.98 1.65 1.58 0.83 1.57 0.44 0.24 0.33 93.54	3.13 0.14 0.07 0.23 5.13 0.56 6.36 40.53 1.76 6.92 2.80 1.38 0.22 0.27 0.19 0.05 0.20 69.93
Hydrodynamic diameter (nm)	78 ± 55^a^ 126 ± 64^b^	80 ± 43^a^ 107 ± 49^b^	68 ± 37^a^ 113 ± 48^b^
Polydispersity index	0.185 ± 0.02^a^ 0.190 ± 0.01^b^	0.455 ± 0.05^a^ 0.470 ± 0.08^b^	0.334 ± 0.06^a^ 0.383 ± 0.02^b^
Zeta potential (mV)	− 22.4 ± 3.22^a^ − 21.5 ± 2.31^b^	− 20.1 ± 1.98^a^ − 19.93 ± 3.98^b^	− 23.5 ± 3.03^a^ − 22.1 ± 2.98^b^

^a^F12+FBS

^b^LHC-9

### DEPs induce single-strand breaks but not oxidative DNA damage in BEAS-2B and A549 cells

**Fig. 1:**
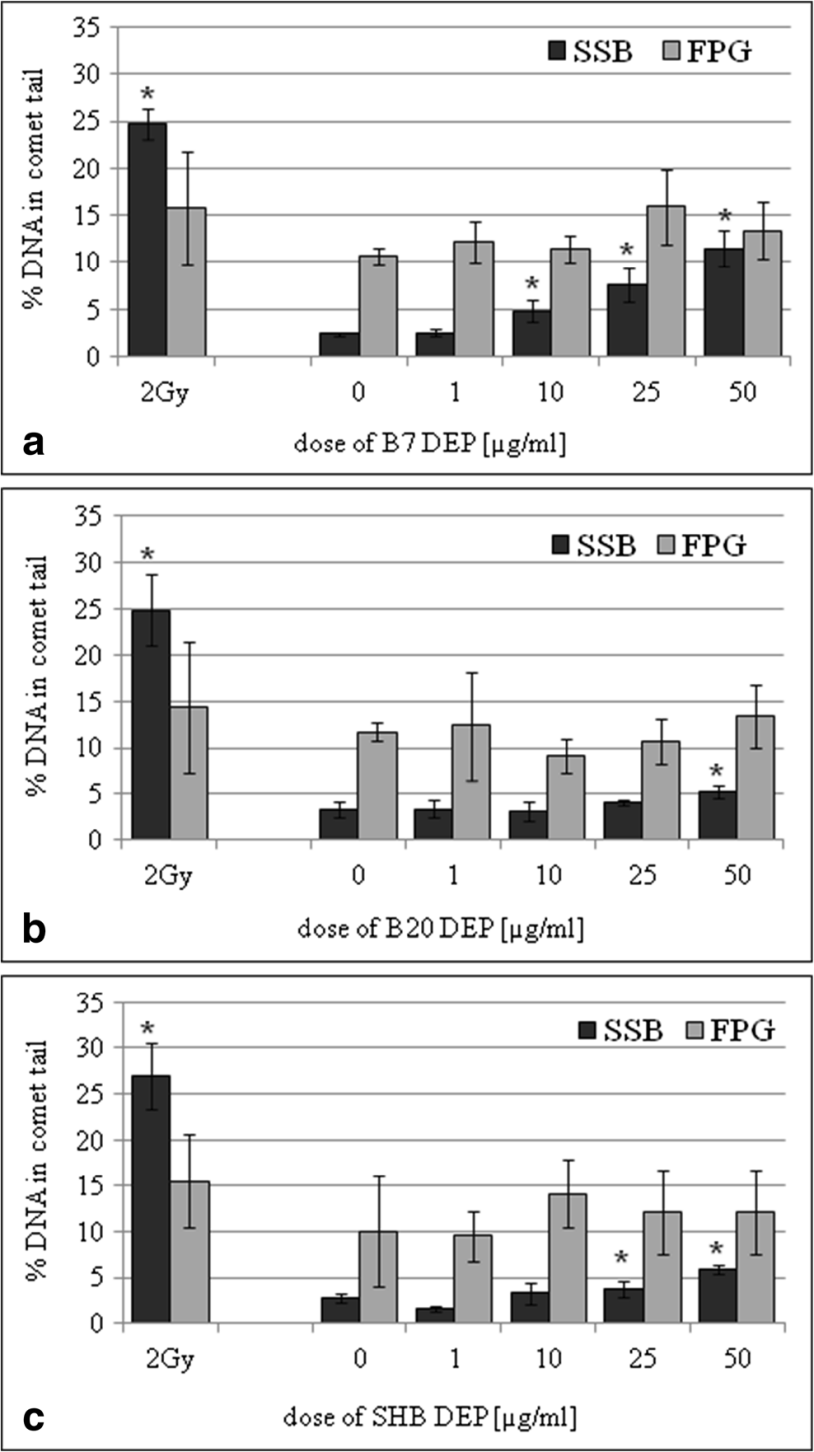
Effect of DEPs on induction of single-strand breaks (SSBs) and oxidative DNA damage (FPG) in BEAS-2B cells. DNA damage was determined by the comet assay. **a** B7-derived DEPs, **b** B20-derived DEPs, and **c** SHB-derived DEPs. Data are expressed as means ± S.D. from three independent experiments. *p* < 0.05. Asterisk denotes statistically significant difference versus corresponding control group

**Fig. 2:**
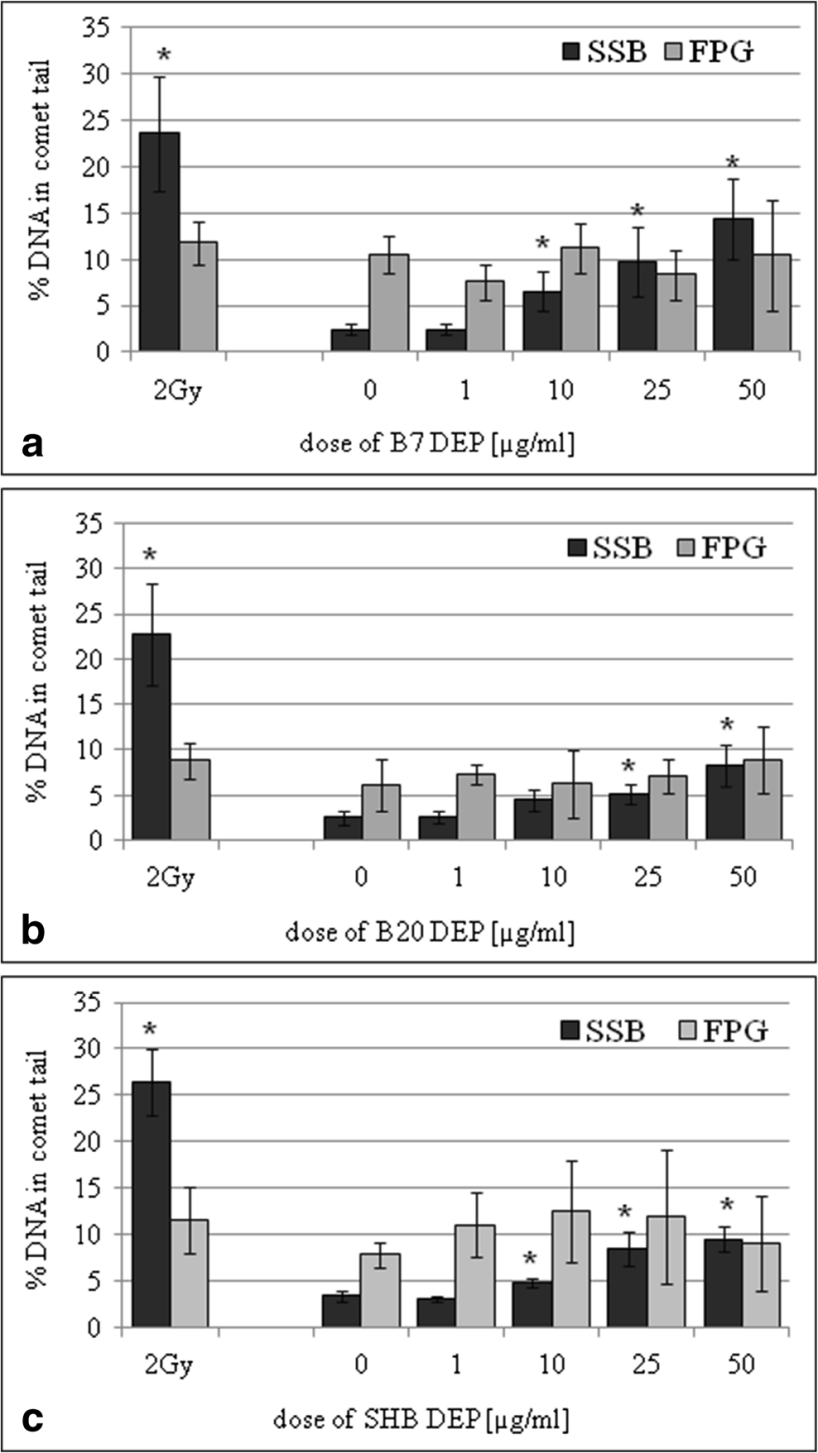
Effect of DEPs on induction of single-strand breaks (SSBs) and oxidative DNA damage (FPG) in A549 cells. DNA damage was determined by the comet assay. **a** B7-derived DEPs, **b** B20-derived DEPs, and **c** SHB-derived DEPs. Data are expressed as means ± S.D. from three independent experiments. *p* < 0.05. Asterisk denotes statistically significant difference versus corresponding control group

BEAS-2B and A549 cells were cultured in the presence of three types of DEPs (1, 10, 25, and 50 μg/mL) for 24 h. DEPs induced DNA damage in BEAS-2B cells versus corresponding control cultures as shown in Fig. 1. The increased level of single-strand DNA breaks (SSBs) was observed in cells incubated with 10, 25, and 50 μg/mL of B7-DEP (*p* = 0.0003, *p* = 0.0001, and *p* = 0.0001, respectively) (Fig. 1a), in cells incubated with 50 μg/mL of B20-DEP (*p* = 0.0036) (Fig. 1b), and in cells incubated with 25 and 50 μg/mL of SHB-DEP (*p* = 0.0002, *p* = 0.0001, respectively). None of the tested DEPs showed significant induction of oxidative DNA damage in BEAS-2B. The effect of DEPs on induction of DNA damage in A549 cells is shown in Fig. 2. The increased level of SSBs was observed in cells incubated with 10, 25, and 50 μg/mL of B7-DEP (*p* = 0.0018, *p* = 0.0001, and *p* = 0.0001, respectively) (Fig. 2a); 25 and 50 μg/mL of B20-DEP (*p* = 0.0383 and *p* = 0.0001, respectively) (Fig. 2b); and in cells incubated with 10, 25, and 50 μg/mL of SHB-DEP (*p* = 0.0301, *p* = 0.0010 and *p* = 0.0001, respectively) (Fig. 2c). None of the tested DEPs showed significant induction of oxidative DNA damage in A549 cells. B7-DEP were the most effective in inducing SSBs in BEAS-2B (*p* = 0.0001 for B7-DEP vs B20-DEP, *p* = 0.0001 for B7-DEP vs SHB-DEP, *p* = 0.2256 for B20-DEP vs SHB-DEP) and A549 cells (*p* = 0.0019 for B7-DEP vs B20-DEP, *p* = 0.0232 for B7-DEP vs SHB-DEP, *p* = 0.3697 for B20-DEP vs SHB-DEP).

### DEPs do not induce double-strand breaks in BEAS-2B and A549 cells

**Fig. 3:**
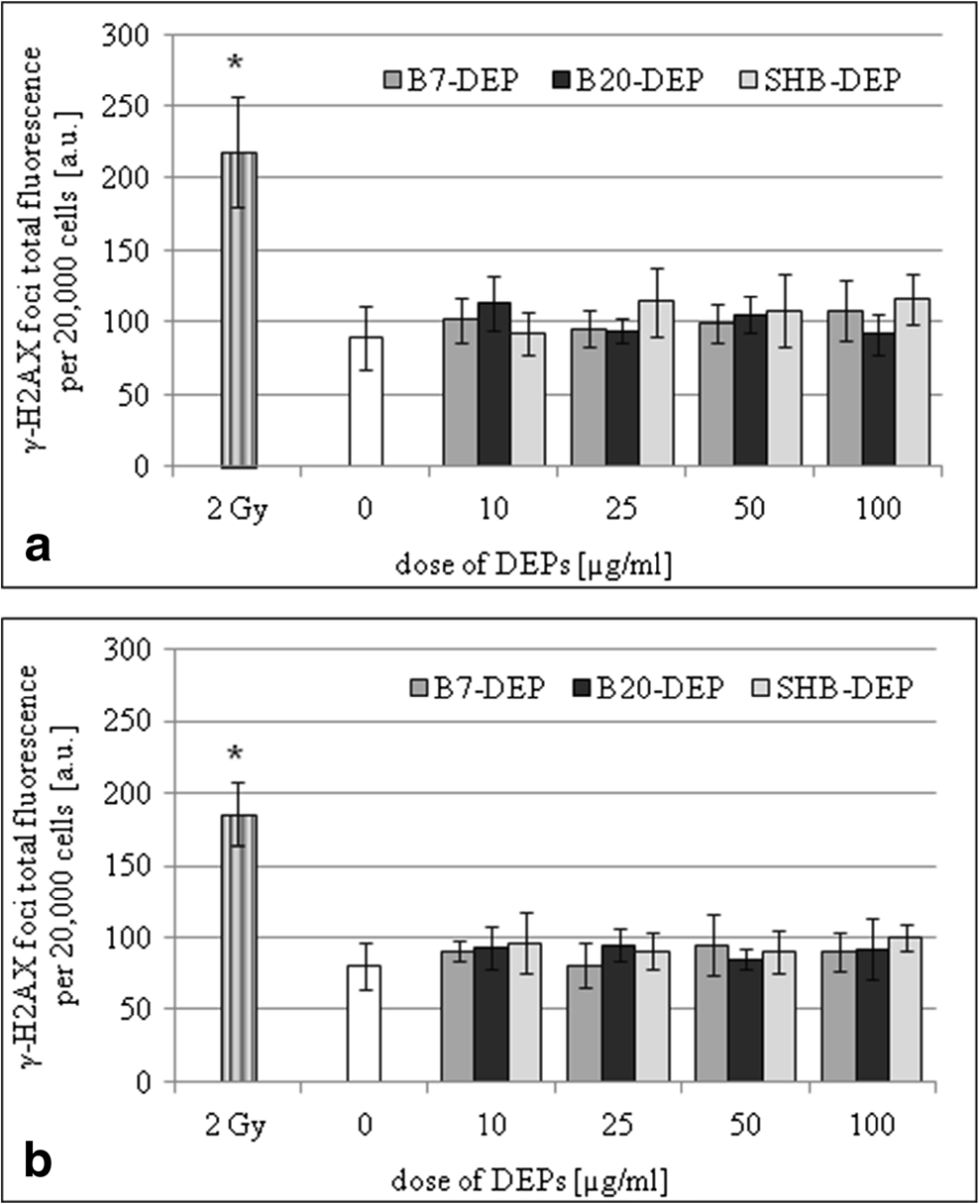
Effect of DEPs (B7, B20, SHB) on induction of double-strand breaks in BEAS-2B (**a**) and A549 cells (**b**). Double-strand breaks were determined by the gamma-H2AX assay. Data are expressed as means ± S.D. from three independent experiments. *p* < 0.05. Asterisk denotes statistically significant difference versus corresponding control group

The gamma-H2AX-detectable double-strand breaks were analyzed in BEAS-2B and A549 cells cultured in the presence of three types of DEPs (10, 25, 50, and 100 μg/mL) for 24 h. As presented in Fig. 3, none of the tested DEPs showed significant induction of the gamma-H2AX foci fluorescence in BEAS-2B (Fig. 3a) and A549 (Fig. 3b) cells. The positive assay control (2 Gy of ionizing radiation) significantly increased the gamma-H2AX foci fluorescence in both cell lines.

### DEPs induce chromosomal damage in BEAS-2B and A549 cells

**Fig. 4:**
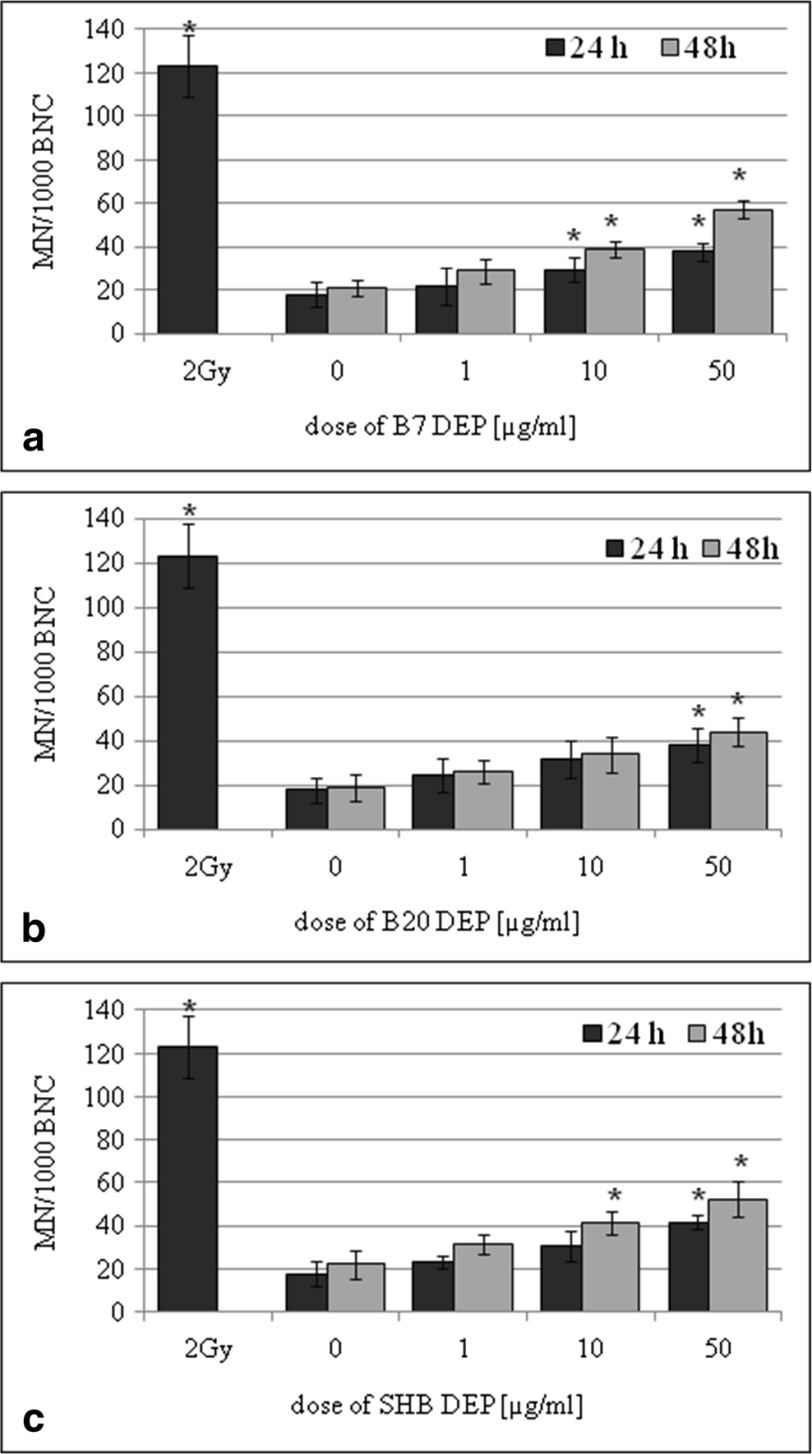
Effect of DEPs on induction of micronuclei in BEAS-2B cells. The frequency of micronuclei (MN) was determined by the micronucleus assay. **a** B7-derived DEPs, **b** B20-derived DEPs, and **c** SHB-derived DEPs. Data are expressed as means ± S.D. from three independent experiments. *p* < 0.05. Asterisk denotes statistically significant difference versus corresponding control group, BNC—binucleated cell

**Fig. 5:**
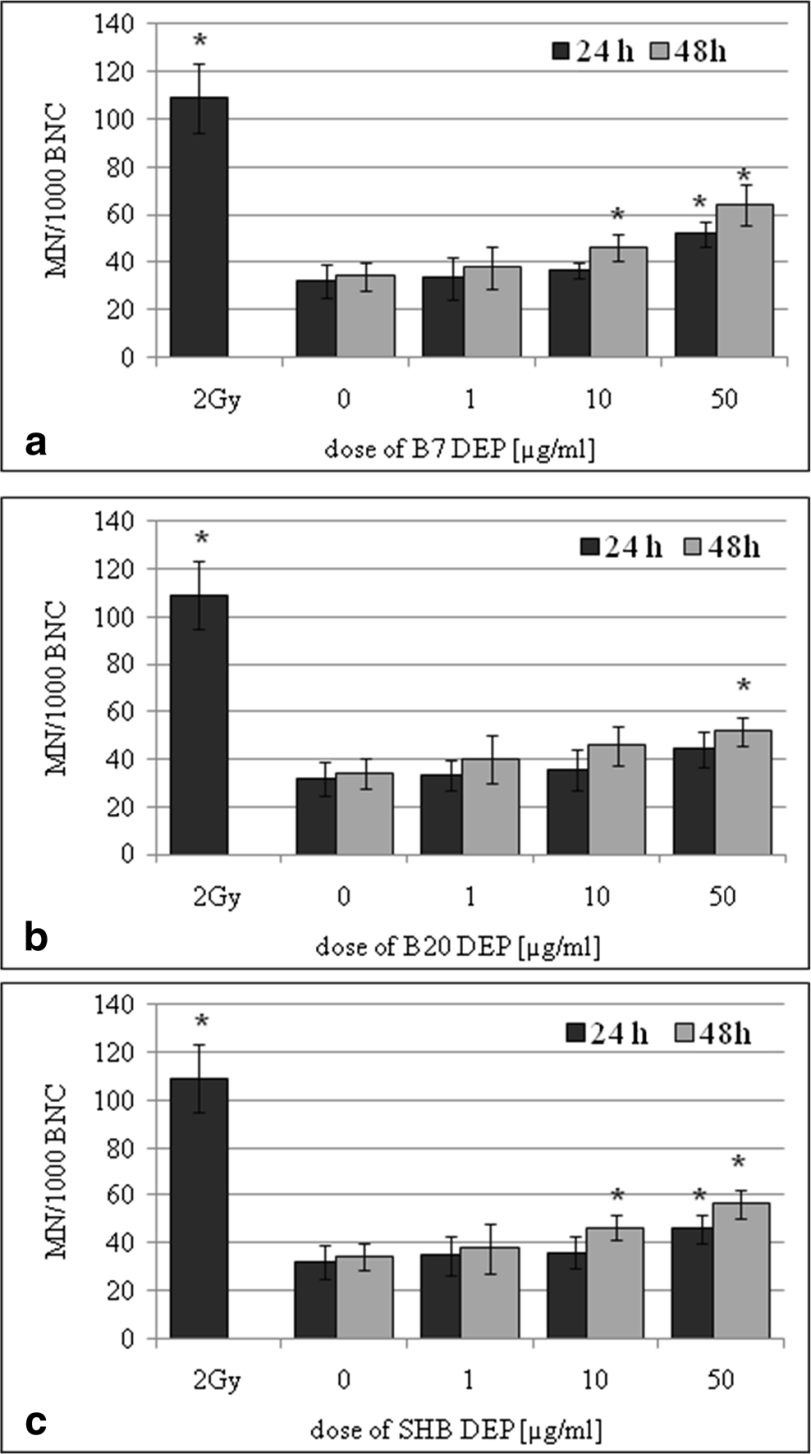
Effect of DEPs on induction of micronuclei in A549 cells. The frequency of micronuclei (MN) was determined following by the micronucleus assay. **a** B7-derived DEPs, **b** B20-derived DEPs, and **c** SHB-derived DEPs. Data are expressed as means ± S.D. from three independent experiments. *p* < 0.05. Asterisk denotes statistically significant difference versus corresponding control group, BNC—binucleated cell

BEAS-2B and A549 cells were cultured in the presence of three types of DEPs (1, 10, and 50 μg/mL) for 24 and 48 h. The effect of DEPs on the frequency of MN in BEAS-2B cells versus corresponding control cultures is shown in Fig. 4. The increased frequency of MN was observed in cultures incubated with 10 and 50 μg/mL of B7-DEP for 24 h (*p* = 0.0177 and *p* = 0.0001, respectively) and for 48 h (*p* = 0.0004 and *p* = 0.0001, respectively) (Fig. 4a). As presented in Fig. 4b, the increased frequency of MN was observed in cultures incubated with 50 μg/mL of B20-DEP for 24 h (*p* = 0.0001) and for 48 h (*p* = 0.0001). As shown in Fig. 4c, the increased frequency of MN was observed in cultures incubated with 50 μg/mL of SHB-DEP for 24 h (*p* = 0.0001) and with 10 and 50 μg/mL for 48 h (*p* = 0.0032, *p* = 0.0016, respectively). The effect of DEPs on the frequency of MN in A549 cells versus corresponding control cultures is shown in Fig. 5. The increased frequency of MN was observed in cultures incubated with 50 μg/mL of B7-DEP for 24 h (*p* = 0.0016) and with 10 and 50 μg/mL for 48 h (*p* = 0.0326 and *p* = 0.0002, respectively) (Fig. 5a). As presented in Fig. 5b, the increased frequency of MN was observed also in cultures incubated with 50 μg/mL of B20-DEP for 48 h (*p* = 0.0032). As shown in Fig. 5c, the increased frequency of MN was observed in cultures incubated with 10 and 50 μg/mL of SHB-DEP for 48 h (*p* = 0.0422 and *p* = 0.0180, respectively). However, statistical analysis revealed no significant difference in inducing micronuclei between three types of DEPs.

### Correlating the alkaline comet assay results with the micronucleus assay results

Correlation of the comet assay results (% DNA in a comet tail 24 h after treatment) with the micronucleus assay results (the frequency of MN in 1000 BNC 24 h after treatment) in BEAS-2B and A549 cells revealed a significant positive correlation between both endpoints. Correlation coefficients for BEAS-2B cells are as follows: for B7-DEP: *r* = 0.9364, *p* = 0.0435; for B20-DEP: *r* = 0.9907, *p* = 0.0382; and for SHB-DEP: *r* = 0.9831, *p* = 0.0292. Correlation coefficients for A549 cells are as follows: for B7-DEP: *r* = 0.9943, *p* = 0.0089; for B20-DEP: *r* = 0.9814, *p* = 0.0382; and for BSHB-DEP: *r* = 0.9648, *p* = 0.0451.

### Gene expression profiling in BEAS-2B and A549 cells exposed to DEPs

**Fig. 6:**
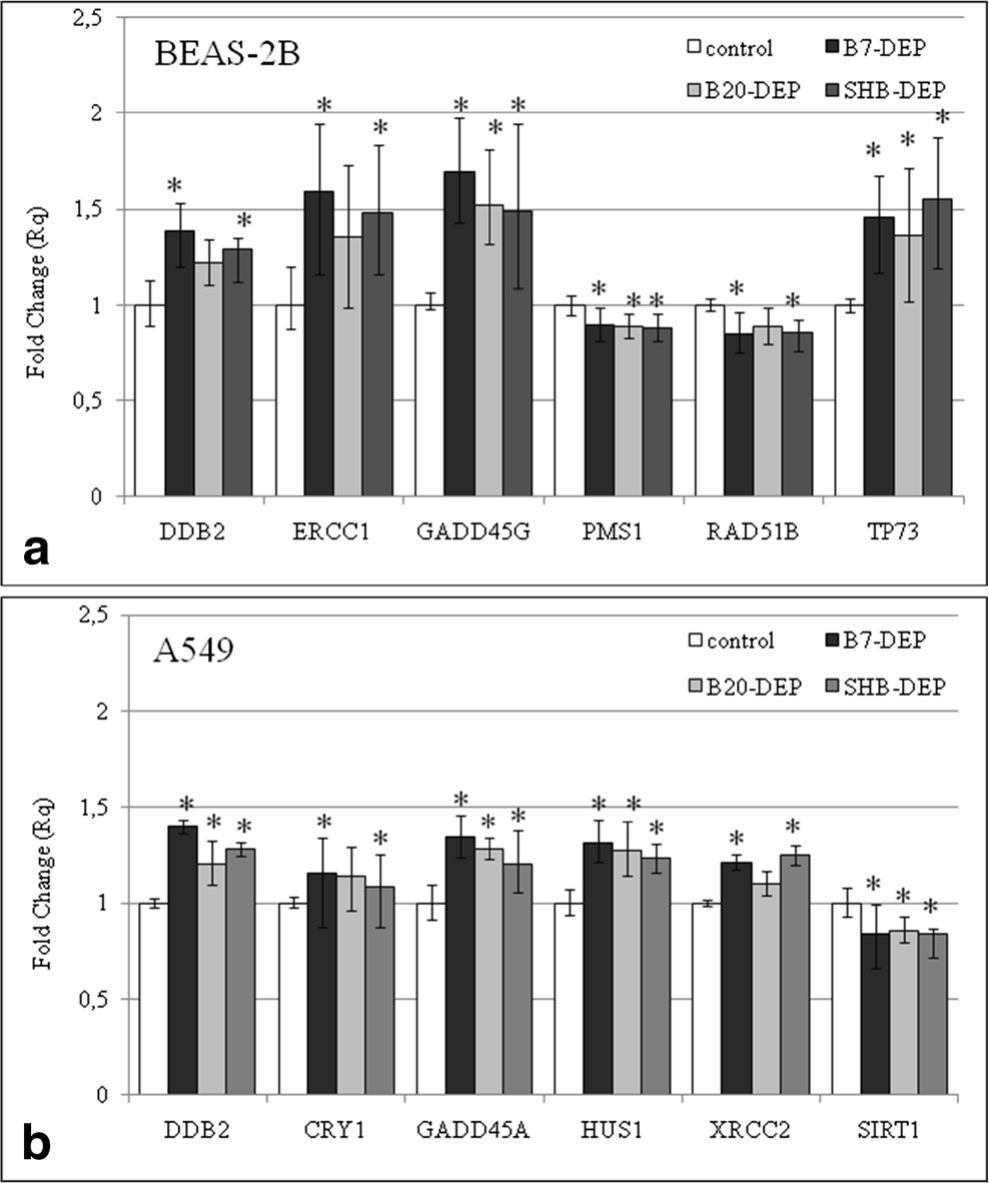
Changes in gene expression in BEAS-2B cells (**a**) and A549 cells (**b**) after treatment with 50 μg/mL of three types of DEPs for 6 h. Mean fold change values from three independent experiments are presented. Error bars represent minimum and maximum values in sample. Fold changes statistically significant in Student’s *t* test are highlighted (asterisk)

To elucidate the molecular mechanism behind the observed effects in BEAS-2B and A549 cells, we analyzed the effects of DEPs on the expression of 84 different genes involved in DNA damage signaling pathways by the real-time PCR (Supplementary Table I and Supplementary Table II). Cells were exposed to 50 μg/mL of DEPs for 6 h. Experiments with BEAS-2B cells revealed that 11 genes appeared to be significantly deregulated by B7-DEP (upregulated: DDB2, ERCC1, GADD45G, and TP73; downregulated: FEN1, MDC1, PMS1, PPM1D, RAD50, RAD51B, and TP53BP1), 4 genes by B20-DEP (upregulated: GADD45G and TP73; downregulated: PMS1, PPM1D), and 11 genes by SHB-DEP (upregulated: DDB2, ERCC1, GADD45G, and TP73; downregulated: FEN1, MDC1, PMS1, PPM1D, RAD50, RAD51B, and TP53BP1) (Fig. 6a). Experiments with A549 cells revealed that 6 genes were significantly deregulated by B7-DEP (upregulated: CRY1, DDB2, GADD45A, HUS1, and XRCC2; downregulated: SIR1), 4 genes by B20-DEP (upregulated: DDB2, GADD45A, and HUS1; downregulated: SIR1), and 6 genes by SHB-DEP (upregulated: CRY1, DDB2, GADD45A, HUS1, and XRCC2; downregulated: SIR1) (Fig. 6b).

## Discussion

Our previous study revealed that particulate emissions from the combustion of two first-generation biofuels, 7% FAME (B7) and 20% FAME (B20), and a second-generation 20% FAME/HVO (SHB) biofuel induced cytotoxic effects in BEAS-2B and A549 cells, manifested as cell death, intracellular reactive oxygen species (ROS) production, and increased expression of antioxidant genes (Lankoff et al. 2017). Since it is commonly accepted that the attack of ROS on DNA may generate a whole series of DNA damage, among them single- and/or double-strand breaks, abasic sites, base and sugar lesions, and a large number of pyrimidine- and purine-derived oxidative DNA lesions (Tudek et al. 2010), the purpose of this study was to determine the genotoxic effects of these particles in BEAS-2B and A549 cells.

Among the available genotoxicity tests, which were assessed by the IARC workgroup with the aim of describing mechanisms of carcinogenicity of air pollution particles, the comet assay (CA) is a widely acknowledged tool in molecular epidemiology and genetic toxicology (Araldi et al. 2015). We applied the alkaline comet assay, which is typically referred to as measuring “DNA SSBs” or “DNA damage.” Our results revealed a dose-dependent increase in the level of SSBs in cells treated with all types of DEPs. Among the studied DEPs, B7-DEP exposure caused maximum DNA damage, while B20-DEP and SHB-DEP induced SSBs at the similar level. This is most likely due to differences in the physicochemical properties of the three types of DEP tested. A number of characteristic parameters of particles affect their toxicity, including their size, shape, surface reactivity, surface charge, surface coating, and elemental composition (Øvrevik et al. 2015). While the size, morphology, and surface charge of the three types of DEPs tested in this study were similar, the presence of organic compounds including PAH and/or nitro-PAH compounds adsorbed onto the particle surface could be expected to have an impact. B7-derived DEP contained the highest concentration of PAHs. The increase in bioadditive ratio caused a decrease in the PAH concentration of sufficient magnitude to diminish the observed effects, as demonstrated for B20-DEPs and SHB-DEPs. The role of PAHs adsorbed on diesel soot emissions has been extensively reviewed, showing that genotoxic effects of organic extracts from combustion-generated particles are mainly connected with PAHs and their derivative (Topinka et al. 2012). Nevertheless, studies related to the associations between biodiesel-derived DEP exposures and the CA endpoints are almost completely missing. Among these reports available, Jalava et al. (2010) used the CA to determine DNA damage in the 264.7 macrophages following exposure to DEPs from the combustion of 100% diesel oil and two biodiesels (100% HVO and 100% RME). They found that all DEP samples induced SSBs at the same level, except weaker response for the RME sample with a catalyst. Two years later, the same group published the results showing that DEPs from the combustion of five fuels (100% diesel, 100% HVO, 30% HVO, 100% RME, 30% RME) induced a dose-dependent fragmentation of chromosomal DNA. Emission particles from the engine powered by 100% diesel and 30% HVO were the most potent inducers of SSBs (Jalava et al. 2012).

One of the commonest sources of SSBs is oxidative attack by endogenous ROS. SSBs can arise directly via disintegration of the oxidized sugar or indirectly during the DNA base excision repair (BER) of oxidized bases and abasic sites, as well as during the nucleotide excision repair (NER) of damaged or altered nucleotides. More recently, it has emerged that SSBs can also arise as a result of erroneous or abortive activity of cellular enzymes such as DNA topoisomerase 1 (Top1) or erroneous incorporation of ribonucleotides into DNA (Caldecott 2014). The alkaline CA does not distinguish between SSBs due to direct oxidative DNA damage and indirect SSBs (Collins et al. 2017). Thus, it has been of particular interest to elucidate the origin of SSBs induced by DEPs tested in our study. We therefore used the enzyme-modified CA, which enable the detection of oxidized bases by combining the assay with the use of a formamidopyrimidine-DNA glycosylase (FPG) converting altered purines into DNA breaks (Collins 2014). We found that none of the tested DEPs showed significant induction of oxidative DNA base damage in BEAS-2B and A549 cells, suggesting the DNA repair-related origin of SSBs rather than oxidative stress-related. On the contrary to our findings, Hemmingsen et al. (2011) found that SSBs and FPG sensitive sites showed concentration-dependent increases in A549 cells exposed to particles from the combustion of RME-derived biofuel (B20) and pure diesel, with small differences between the B20 and B0 particles. However, along with our study, Gualtieri et al. (2011) reported that urban PM2.5 were more potent compared to PM10 for SSB generation in BEAS-2B cells, whereas there was no effect on FPG total sites. On the contrary, Jantzen et al. (2012) reported that the reference DEPs (SRM2975 and SRM1650b) generated SSBs and oxidatively damaged DNA, measured using the enzyme-modified CA on formamidopyrimidine-DNA glycosylase or oxoguanine DNA glycosylase (hOGG1)-sensitive sites in A549 and THP-1a cells, as well as in co-cultures of A549 and THP-1a cells. Recently, Vattanasit et al. (2014) observed the dose-dependent oxidative DNA damage in the lymphoblasts and lung cells exposed to DEPs.

Single-strand breaks can have an impact on cell fate, if they are not repaired rapidly and appropriately. The most likely consequence of the increased level of SSBs in non-proliferating cells is cell death by stalling of RNA polymerases during transcription. However, in proliferating cells, the most likely consequence is the blockage or collapse of DNA replication forks during the S phase of the cell cycle, leading to the formation of double-strand breaks (DSBs), chromosome rearrangements, and genomic instability (Woodbine et al. 2011). To elucidate whether the increased level of SSBs observed in the comet assay results in induction of DSBs, we treated BEAS-2B and A549 cells in the same manner as for the analysis of SSBs and used the γ-H2AX assay, which is considered attributable to the direct formation of DSBs (Rogakou et al. 1998). Our results revealed that none of the tested DEPs showed induction of the gamma-H2AX-detectable double-strand breaks. To the best of our knowledge, a study investigating the effects of biodiesel-derived DEPs or their organic extracts on induction of DSBs has not been published yet, so comparable data are not available. However, Toyooka et al. (2012) determined the formation of DSBs in A549, MCF7, HaCaT, and A549 cells exposed to 9,10-phenanthrenequinone (9,10-PQ), a major quinone in DEPs, and reported small amounts of γ-H2AX, shown as fluorescence foci.

To further elucidate whether the increased level of SSBs observed in the comet assay may be related to DNA damage at the chromosome level, we applied the micronucleus assay, a multi-target genotoxic endpoint, assessing not only clastogenic and aneugenic events but also some epigenetic effects (Kirsch-Volders et al. 2011). Our results revealed that all types of DEPs increased the frequency of micronuclei in a dose- and time-dependent manner in BEAS-2B and A549 cells when compared to controls. However, statistical analysis revealed no significant difference in inducing micronuclei between the three types of DEPs. Our results are in line with the findings of Cervena et al. (2016), who observed significantly increased frequency of MN in BEAS-2B cells exposed to extractable organic matter from particle emissions from combustion of various biodiesel fuels and pure diesels (B0, B30, B100). The genotoxicity of these extracts was comparable. On the contrary, Leme et al. (2012) studied the genotoxic mode of action of pure soybean biodiesel water extract (B100) and its blends in diesel oil (B5, B20, B50) using the flow cytometry-based MN assay. These authors reported a clear increase in the MN frequency after exposure of CHO-K1 to the B100 sample and suggested a clastogenic mode of action. Apart from the B100 sample, no other test extract showed significant MN induction in this test system.

Comparative analysis of genotoxicity of the three types of DEPs in BEAS-2B and A549 cells by the alkaline CA, the enzyme-modified CA, the gamma-H2AX assay, and the MN assay showed significant positive correlations between endpoints evaluated by the alkaline CA and the MN assay. The overall results suggest that the increased level of SSBs is likely the indicator of DNA damage induction and repair due to the presence of breaks in the lesion repair via BER or NER. To confirm our assumption, we analyzed the expression of 84 different genes involved in DNA damage signaling pathways by the real-time PCR. Supplementary Table I summarizes the differential expression of the genes tested in BEAS-2B cells. Among these genes, commonly upregulated genes were as follows: DDB2, ERCC1, TP73, and GADD45G. Elevated expression of DDB2 (damaged DNA binding protein 2) may be interpreted as response to DNA damage and repair, since this gene encodes a smaller subunit of the damaged DNA binding protein DDB, which recognizes DNA damage and is required for efficient NER, a DNA repair pathway involved in the removal of bulky DNA adducts (Forestier et al. 2015). ERCC1 (DNA excision repair protein 1) forms the ERCC1-XPF enzyme complex that participates in NER and repair of inter-strand crosslinks (Formica et al. 2017). Upregulated expression of TP73 (p73) is an indicator of DNA damage as TP73 promotes a growth arrest and/or apoptosis similar to p53 (Candi et al. 2014). GADD45G (the growth arrest and DNA damage-inducible 45) protein plays an important role in cellular genotoxic and non-genotoxic stress responses including NER and cell cycle control (Hildesheim et al. 2002). In addition, downregulation of FEN1, MDC1, PMS1, PPM1D, RAD50, and TP53BP1 may suggest disturbances of DNA damage recognition and repair since FEN1 (flap endonuclease 1) removes 5′ overhanging “flaps” of single-stranded DNA (Balakrishnan and Bambara 2013); MDC1 (mediator of DNA damage checkpoint protein 1) is part of the DNA damage response pathway, the mechanism through which cells respond to damaged DNA (Coster and Goldberg 2010); PPP1R15A **(**protein phosphatase 1 regulatory subunit 15A) responds to treatment with DNA-damaging agents; PMS1 (PMS1 protein homolog 1) is involved in DNA mismatch repair (Goellner et al. 2015); PPM1D (Protein phosphatase 1D) is a negative regulator of cell stress response pathways (Zhu and Bulavin 2012); RAD50 (DNA repair protein RAD50) is involved in DSBs repair; and TP53BP1 (tumor suppressor p53-binding protein 1) plays a key role in response to DNA damage (Panier and Boulton 2014). We also identified several genes in A549 cells that were differentially expressed as compared to BEAS-2B cells (Supplementary Table II). Among these genes, commonly upregulated genes were as follows: HUS1 and XRCC2. Elevated expression of HUS1 (checkpoint protein HUS1**)** may suggest response to DNA damage since the protein encoded by this gene is a component of genotoxin-activated checkpoint complex that is involved in the cell cycle arrest in response to DNA damage (Weiss et al. 2000). XRCC2 (DNA repair protein XRCC2**)** is involved in the repair of DNA double-strand breaks by homologous recombination (Thacker and Zdzienicka 2004). However, SIR1 (sirtuin 1) is an intracellular regulatory protein with mono-ADP-ribosyltransferase activity (Abdellatif 2012). Taken together, these data clearly indicate that all types of DEPs deregulated expression of genes, which encode proteins playing an important role in response to DNA damage and repair in BEAS-2B and A549 cells. These proteins are mainly involved in recognition of DNA damage, cell cycle arrest in response to DNA damage, and its repair by NER, a DNA repair pathway involved in the removal of bulky DNA adducts. Therefore, the gene expression results support our assumption that the increased level of SSBs is likely the indicator of DNA repair due to the presence of breaks in the lesion repair via NER.

## Conclusions

To conclude, our findings indicate that particulate engine emissions from each type of biodiesel fuel induced genotoxic effects in BEAS-2B and A549 cells, manifested either as the increased levels of single-strand breaks, the increased frequencies of micronuclei, or deregulated expression of genes involved in DNA damage signaling pathways. Our results revealed also that none of the tested DEPs caused the induction of oxidative DNA damage and the gamma-H2AX-detectable double-strand breaks. The most pronounced differences concerning the tested particles were observed for the induction of single-strand breaks, with the greatest genotoxicity being associated with the B7-derived DEPs. Differences in other effects between DEP from the different biodiesel blend percentage and biodiesel feedstock were also observed, but the magnitude of these differences were rather marginal. Overall, this suggests that increasing the concentration of FAME in biodiesel from the current 7 to 20% or substituting FAME with HVO affects the toxicity from DEP emissions, but the biological significance of this may be moderate. However, these results should be taken with some caution, since they were obtained in in vitro systems. A combination of these results with the results from in vivo genotoxicity studies, performed as part of the FuelHealth project (unpublished results), should help to better understand the toxicity induced by DEPs from the combustion of various biodiesel fuels.

## Electronic supplementary material

Supplementary Table I (DOCX 135 kb)

Supplementary Table II (DOCX 134 kb)
