# Supplementary material for: Genotoxic potential of diesel exhaust particles from the combustion of first- and second-generation biodiesel fuels—the FuelHealth project
Source: Environ Sci Pollut Res Int. 2017 Sep 9;24(31):24223–34. doi: 10.1007/s11356-017-9995-0 (PMC5655577; doi:10.1007/s11356-017-9995-0)
Supplement: Supplementary file 1 — (DOCX 135 kb) [file 11356_2017_9995_MOESM1_ESM.docx]

**Supplementary Table I.** Changes in gene expression in BEAS-2B cells after treatment with 50 µg/ml of three types of DEPs (B7-derived DEPs, B20-derived DEPs and SHB-derived DEPs) for 6 hours. Mean fold change values from three independent experiments are presented. Fold changes statistically significant in Student’s t-test are highlighted in green (up-regulated genes) or red (down-regulated genes).

| **Target Name** | **UniGene** | **B20-DEPs** | | **B7-DEPs** | | **SHB-DEPs** | |
| --- | --- | --- | --- | --- | --- | --- | --- |
|  |  | **Mean fold change** | **t-test**  **p-value** | **Mean fold change** | **t-test**  **p-value** | **Mean fold change** | **t-test**  **p-value** |
| ABL1 | Hs.431048 | 0,991 | 0,918 | 1,07 | 0,476 | 1,01 | 0,907 |
| APEX1 | Hs.73722 | 1,016 | 0,808 | 1,04 | 0,364 | 1,023 | 0,599 |
| ATM | Hs.367437 | 0,924 | 0,466 | 0,99 | 0,928 | 0,946 | 0,603 |
| ATR | Hs.271791 | 0,895 | 0,135 | 0,883 | 0,130 | 0,887 | 0,231 |
| ATRIP | Hs.694840 | 1,017 | 0,773 | 1,099 | 0,174 | 1,026 | 0,666 |
| ATRX | Hs.533526 | 0,956 | 0,4 | 0,905 | 0,121 | 0,91 | 0,137 |
| BARD1 | Hs.591642 | 0,973 | 0,761 | 0,947 | 0,556 | 0,965 | 0,694 |
| BAX | Hs.624291 | 1,008 | 0,932 | 1,196 | 0,143 | 1,081 | 0,444 |
| BBC3 | Hs.467020 | 0,994 | 0,948 | 1,196 | 0,304 | 0,994 | 0,945 |
| BLM | Hs.725208 | 0,975 | 0,585 | 1,003 | 0,897 | 0,94 | 0,087 |
| BRCA1 | Hs.194143 | 0,963 | 0,183 | 0,994 | 0,836 | 0,946 | 0,123 |
| BRIP1 | Hs.128903 | 0,913 | 0,154 | 0,919 | 0,189 | 0,923 | 0,242 |
| CDC25A | Hs.437705 | 0,945 | 0,46 | 1,054 | 0,472 | 0,983 | 0,82 |
| CDC25C | Hs.656 | 0,972 | 0,644 | 1,084 | 0,48 | 1,01 | 0,756 |
| CDK7 | Hs.184298 | 0,994 | 0,866 | 0,95 | 0,323 | 0,956 | 0,172 |
| CDKN1A | Hs.370771 | 1,013 | 0,561 | 0,919 | 0,005 | 0,93 | 0,025 |
| CHEK1 | Hs.24529 | 0,959 | 0,283 | 0,996 | 0,915 | 0,929 | 0,12 |
| CHEK2 | Hs.291363 | 1,022 | 0,705 | 1,008 | 0,89 | 1,01 | 0,874 |
| CIB1 | Hs.715556 | 1,018 | 0,763 | 1,013 | 0,834 | 0,981 | 0,725 |
| CRY1 | Hs.151573 | 0,902 | 0,191 | 0,943 | 0,427 | 0,902 | 0,207 |
| CSNK2A2 | Hs.82201 | 0,934 | 0,303 | 0,977 | 0,696 | 0,914 | 0,205 |
| DDB1 | Hs.290758 | 0,965 | 0,5 | 1,108 | 0,165 | 1,077 | 0,248 |
| DDB2 | Hs.700338 | 0,916 | 0,098 | 1,389 | 0,032 | 1,287 | 0,036 |
| DDIT3 | Hs.505777 | 1,006 | 0,89 | 0,964 | 0,356 | 0,936 | 0,179 |
| ERCC1 | Hs.435981 | 1,356 | 0,134 | 1,59 | 0,039 | 1,482 | 0,044 |
| ERCC2 | Hs.487294 | 1,009 | 0,89 | 1,19 | 0,065 | 1,116 | 0,116 |
| EXO1 | Hs.498248 | 1,006 | 0,912 | 0,966 | 0,552 | 0,941 | 0,383 |
| FANCA | Hs.744083 | 0,967 | 0,705 | 1,007 | 0,943 | 0,947 | 0,548 |
| FANCD2 | Hs.208388 | 0,924 | 0,141 | 1,003 | 0,939 | 0,925 | 0,121 |
| FANCG | Hs.591084 | 0,922 | 0,326 | 1,004 | 0,961 | 0,956 | 0,514 |
| FEN1 | Hs.409065 | 0,981 | 0,586 | 0,914 | 0,023 | 0,912 | 0,022 |
| GADD45A | Hs.80409 | 1,029 | 0,834 | 1,082 | 0,483 | 1,074 | 0,615 |
| GADD45G | Hs.9701 | 1,696 | 0,036 | 1,522 | 0,028 | 1,488 | 0,035 |
| H2AFX | Hs.477879 | 0,967 | 0,284 | 1,122 | 0,065 | 1,009 | 0,844 |
| HUS1 | Hs.152983 | 0,921 | 0,541 | 1,216 | 0,115 | 1,162 | 0,192 |
| LIG1 | Hs.1770 | 0,97 | 0,717 | 1,042 | 0,693 | 0,998 | 0,986 |
| MAPK12 | Hs.432642 | 0,997 | 0,983 | 1,255 | 0,262 | 1,15 | 0,34 |
| MBD4 | Hs.35947 | 1,012 | 0,767 | 1,032 | 0,443 | 1,004 | 0,918 |
| MCPH1 | Hs.593807 | 0,984 | 0,826 | 1,049 | 0,512 | 0,979 | 0,735 |
| MDC1 | Hs.653495 | 0,971 | 0,289 | 0,946 | 0,038 | 0,919 | 0,025 |
| MLH1 | Hs.195364 | 1,013 | 0,795 | 1,009 | 0,86 | 0,987 | 0,796 |
| MLH3 | Hs.436650 | 0,957 | 0,641 | 0,97 | 0,737 | 0,904 | 0,277 |
| MPG | Hs.459596 | 1,019 | 0,854 | 1,028 | 0,788 | 1,061 | 0,449 |
| MRE11A | Hs.192649 | 0,94 | 0,333 | 0,964 | 0,514 | 0,919 | 0,223 |
| MSH2 | Hs.597656 | 1,014 | 0,69 | 0,961 | 0,281 | 0,924 | 0,081 |
| MSH3 | Hs.648635 | 0,923 | 0,228 | 0,945 | 0,238 | 0,95 | 0,282 |
| NBN | Hs.492208 | 0,971 | 0,508 | 1,022 | 0,636 | 0,994 | 0,881 |
| NTHL1 | Hs.66196 | 0,979 | 0,655 | 0,96 | 0,065 | 0,957 | 0,201 |
| OGG1 | Hs.380271 | 1 | 0,997 | 1,099 | 0,461 | 1,066 | 0,56 |
| PARP1 | Hs.177766 | 1,018 | 0,885 | 1,176 | 0,201 | 1,112 | 0,37 |
| PCNA | Hs.147433 | 0,979 | 0,375 | 0,949 | 0,097 | 0,937 | 0,052 |
| PMS1 | Hs.111749 | 0,888 | 0,005 | 0,821 | 0,004 | 0,859 | 0,009 |
| PMS2 | Hs.715590 | 0,908 | 0,092 | 0,919 | 0,112 | 0,895 | 0,055 |
| PNKP | Hs.78016 | 1 | 0,998 | 1,25 | 0,223 | 1,089 | 0,502 |
| PPM1D | Hs.286073 | 0,886 | 0,031 | 0,896 | 0,043 | 0,88 | 0,026 |
| PPP1R15A | Hs.631593 | 1,092 | 0,519 | 1,167 | 0,381 | 1,065 | 0,638 |
| PRKDC | Hs.491682 | 0,981 | 0,677 | 0,914 | 0,066 | 0,939 | 0,079 |
| RAD1 | Hs.38114 | 0,987 | 0,567 | 0,998 | 0,943 | 0,968 | 0,204 |
| RAD17 | Hs.16184 | 0,967 | 0,444 | 0,963 | 0,449 | 0,933 | 0,182 |
| RAD18 | Hs.375684 | 0,943 | 0,064 | 0,957 | 0,203 | 0,922 | 0,051 |
| RAD21 | Hs.81848 | 0,992 | 0,801 | 1,104 | 0,113 | 1,055 | 0,234 |
| RAD50 | Hs.633509 | 0,916 | 0,163 | 0,884 | 0,049 | 0,893 | 0,011 |
| RAD51 | Hs.631709 | 0,974 | 0,571 | 0,943 | 0,213 | 0,936 | 0,176 |
| RAD51B | Hs.172587 | 0,884 | 0,115 | 0,848 | 0,025 | 0,854 | 0,002 |
| RAD9A | Hs.655354 | 0,888 | 0,364 | 1,075 | 0,543 | 1,027 | 0,84 |
| RBBP8 | Hs.546282 | 0,983 | 0,694 | 0,909 | 0,196 | 0,927 | 0,102 |
| REV1 | Hs.443077 | 0,92 | 0,294 | 0,976 | 0,719 | 0,893 | 0,178 |
| RNF168 | Hs.250648 | 0,967 | 0,782 | 1,228 | 0,177 | 1,091 | 0,493 |
| RNF8 | Hs.485278 | 0,95 | 0,234 | 1,051 | 0,275 | 0,977 | 0,631 |
| RPA1 | Hs.461925 | 0,972 | 0,526 | 1,007 | 0,848 | 0,975 | 0,487 |
| SIRT1 | Hs.369779 | 0,943 | 0,079 | 0,962 | 0,28 | 0,926 | 0,091 |
| SMC1A | Hs.211602 | 0,993 | 0,83 | 1,047 | 0,358 | 1,009 | 0,787 |
| SUMO1 | Hs.81424 | 1,033 | 0,663 | 1,05 | 0,52 | 1,066 | 0,407 |
| TOPBP1 | Hs.593379 | 0,96 | 0,229 | 0,997 | 0,912 | 0,952 | 0,152 |
| TP53 | Hs.437460 | 0,952 | 0,397 | 0,991 | 0,733 | 0,962 | 0,283 |
| TP53BP1 | Hs.440968 | 0,952 | 0,122 | 0,812 | 0,027 | 0,794 | 0,017 |
| TP73 | Hs.192132 | 1,365 | 0,047 | 1,456 | 0,026 | 1,556 | 0,028 |
| UNG | Hs.191334 | 0,93 | 0,167 | 0,895 | 0,145 | 0,86 | 0,127 |
| XPA | Hs.654364 | 0,826 | 0,237 | 1,046 | 0,454 | 0,892 | 0,174 |
| XPC | Hs.475538 | 0,861 | 0,512 | 1,218 | 0,418 | 0,95 | 0,812 |
| XRCC1 | Hs.98493 | 1,006 | 0,925 | 1,065 | 0,487 | 1,059 | 0,321 |
| XRCC2 | Hs.647093 | 0,95 | 0,577 | 1,068 | 0,315 | 1,066 | 0,32 |
| XRCC3 | Hs.592325 | 1,019 | 0,812 | 1,222 | 0,373 | 1,062 | 0,545 |
| XRCC6 | Hs.292493 | 0,993 | 0,898 | 1,017 | 0,753 | 0,961 | 0,464 |
