# Supplementary material for: Genotoxic potential of diesel exhaust particles from the combustion of first- and second-generation biodiesel fuels—the FuelHealth project
Source: Environ Sci Pollut Res Int. 2017 Sep 9;24(31):24223–34. doi: 10.1007/s11356-017-9995-0 (PMC5655577; doi:10.1007/s11356-017-9995-0)
Supplement: Supplementary file 2 — (DOCX 134 kb) [file 11356_2017_9995_MOESM2_ESM.docx]

**Supplementary Table II.** Changes in gene expression in A549 cells after treatment with 50 µg/ml of three types of DEPs (B7-derived DEPs, B20-derived DEPs and SHB-derived DEPs) for 6 hours. Mean fold change values from three independent experiments are presented. Fold changes statistically significant in Student’s t-test are highlighted in green (up-regulated genes) or red (down-regulated genes).

| **Target Name** | **UniGene** | **B20-DEPs** | | **B7-DEPs** | | **SHB-DEPs** | |
| --- | --- | --- | --- | --- | --- | --- | --- |
|  |  | **Mean fold change** | **t-test**  **p-value** | **Mean fold change** | **t-test**  **p-value** | **Mean fold change** | **t-test**  **p-value** |
| ABL1 | Hs.431048 | 1 | 0,995 | 0,971 | 0,632 | 1,03 | 0,601 |
| APEX1 | Hs.73722 | 1,031 | 0,472 | 1,007 | 0,733 | 1,237 | 0,108 |
| ATM | Hs.367437 | 1,064 | 0,348 | 1,012 | 0,872 | 1,032 | 0,48 |
| ATR | Hs.271791 | 0,964 | 0,544 | 1,009 | 0,913 | 0,947 | 0,38 |
| ATRIP | Hs.694840 | 1,005 | 0,919 | 1,048 | 0,313 | 1,023 | 0,688 |
| ATRX | Hs.533526 | 1,083 | 0,14 | 1,045 | 0,357 | 1,027 | 0,398 |
| BARD1 | Hs.591642 | 1,088 | 0,102 | 1,059 | 0,201 | 1,021 | 0,704 |
| BAX | Hs.624291 | 0,958 | 0,65 | 0,974 | 0,759 | 1,011 | 0,898 |
| BBC3 | Hs.467020 | 0,74 | 0,229 | 0,643 | 0,125 | 0,86 | 0,512 |
| BLM | Hs.725208 | 1,092 | 0,181 | 1,068 | 0,255 | 1,109 | 0,125 |
| BRCA1 | Hs.194143 | 1,014 | 0,607 | 1,003 | 0,881 | 0,99 | 0,484 |
| BRIP1 | Hs.128903 | 1,044 | 0,592 | 1,055 | 0,5 | 1,005 | 0,944 |
| CDC25A | Hs.437705 | 1,037 | 0,724 | 0,996 | 0,971 | 1,026 | 0,781 |
| CDC25C | Hs.656 | 0,977 | 0,547 | 0,927 | 0,127 | 1,003 | 0,926 |
| CDK7 | Hs.184298 | 0,972 | 0,343 | 1 | 0,992 | 0,994 | 0,855 |
| CDKN1A | Hs.370771 | 0,963 | 0,556 | 1,111 | 0,277 | 0,935 | 0,357 |
| CHEK1 | Hs.24529 | 1,022 | 0,345 | 1,006 | 0,876 | 1,004 | 0,888 |
| CHEK2 | Hs.291363 | 0,972 | 0,588 | 0,942 | 0,282 | 0,963 | 0,444 |
| CIB1 | Hs.715556 | 0,966 | 0,373 | 0,952 | 0,22 | 0,955 | 0,326 |
| CRY1 | Hs.151573 | 1,141 | 0,138 | 1,152 | 0,004 | 1,183 | 0,026 |
| CSNK2A2 | Hs.82201 | 1,012 | 0,709 | 1,021 | 0,349 | 1,011 | 0,58 |
| DDB1 | Hs.290758 | 1,032 | 0,463 | 0,969 | 0,464 | 1,041 | 0,409 |
| DDB2 | Hs.700338 | 1,204 | 0,039 | 1,398 | 0,032 | 1,281 | 0,016 |
| DDIT3 | Hs.505777 | 0,784 | 0,204 | 0,721 | 0,228 | 0,894 | 0,502 |
| ERCC1 | Hs.435981 | 1,006 | 0,923 | 0,971 | 0,667 | 1,05 | 0,467 |
| ERCC2 | Hs.487294 | 1,011 | 0,943 | 1,000 | 0,999 | 1,076 | 0,626 |
| EXO1 | Hs.498248 | 1,041 | 0,302 | 0,995 | 0,795 | 1,023 | 0,435 |
| FANCA | Hs.744083 | 0,961 | 0,756 | 0,931 | 0,588 | 0,996 | 0,976 |
| FANCD2 | Hs.208388 | 1,071 | 0,125 | 1,055 | 0,088 | 1,057 | 0,092 |
| FANCG | Hs.591084 | 1,013 | 0,882 | 0,972 | 0,735 | 1,067 | 0,5 |
| FEN1 | Hs.409065 | 1,106 | 0,223 | 1,094 | 0,13 | 1,068 | 0,326 |
| GADD45A | Hs.80409 | 1,283 | 0,029 | 1,344 | 0,015 | 1,205 | 0,013 |
| GADD45G | Hs.9701 | 0,989 | 0,961 | 1,265 | 0,144 | 1,086 | 0,577 |
| H2AFX | Hs.477879 | 0,942 | 0,674 | 0,91 | 0,538 | 0,995 | 0,974 |
| HUS1 | Hs.152983 | 1,275 | 0,043 | 1,316 | 0,013 | 1,232 | 0,015 |
| LIG1 | Hs.1770 | 1,039 | 0,237 | 1,046 | 0,095 | 1,098 | 0,056 |
| MAPK12 | Hs.432642 | 0,893 | 0,365 | 0,873 | 0,312 | 0,975 | 0,823 |
| MBD4 | Hs.35947 | 0,947 | 0,149 | 0,941 | 0,165 | 0,916 | 0,055 |
| MCPH1 | Hs.593807 | 1,033 | 0,507 | 1,054 | 0,215 | 1,019 | 0,406 |
| MDC1 | Hs.653495 | 1,064 | 0,394 | 1,061 | 0,318 | 1,066 | 0,257 |
| MLH1 | Hs.195364 | 1,039 | 0,308 | 1,003 | 0,931 | 1,011 | 0,699 |
| MLH3 | Hs.436650 | 1,094 | 0,086 | 1,118 | 0,059 | 1,099 | 0,089 |
| MPG | Hs.459596 | 1,053 | 0,273 | 1,066 | 0,223 | 1,052 | 0,323 |
| MRE11A | Hs.192649 | 1,056 | 0,25 | 1,016 | 0,712 | 1,045 | 0,327 |
| MSH2 | Hs.597656 | 1,095 | 0,292 | 1,071 | 0,387 | 1,048 | 0,553 |
| MSH3 | Hs.648635 | 1,022 | 0,54 | 1,024 | 0,578 | 1,012 | 0,637 |
| NBN | Hs.492208 | 0,984 | 0,477 | 1,015 | 0,559 | 0,969 | 0,322 |
| NTHL1 | Hs.66196 | 1,06 | 0,098 | 1,066 | 0,205 | 1,069 | 0,182 |
| OGG1 | Hs.380271 | 0,963 | 0,629 | 0,984 | 0,828 | 1,002 | 0,981 |
| PARP1 | Hs.177766 | 1,054 | 0,342 | 1,031 | 0,417 | 1,069 | 0,127 |
| PCNA | Hs.147433 | 0,986 | 0,712 | 0,971 | 0,501 | 0,945 | 0,215 |
| PMS1 | Hs.111749 | 1,006 | 0,832 | 1,007 | 0,789 | 0,962 | 0,524 |
| PMS2 | Hs.715590 | 1,003 | 0,968 | 1,026 | 0,74 | 1,046 | 0,567 |
| PNKP | Hs.78016 | 0,833 | 0,366 | 0,814 | 0,32 | 0,949 | 0,782 |
| PPM1D | Hs.286073 | 0,98 | 0,415 | 0,962 | 0,522 | 0,975 | 0,466 |
| PPP1R15A | Hs.631593 | 0,928 | 0,681 | 0,904 | 0,614 | 1,015 | 0,935 |
| PRKDC | Hs.491682 | 1,184 | 0,162 | 1,234 | 0,045 | 1,126 | 0,06 |
| RAD1 | Hs.38114 | 1,006 | 0,901 | 1,024 | 0,672 | 0,973 | 0,693 |
| RAD17 | Hs.16184 | 0,958 | 0,257 | 1,028 | 0,634 | 0,955 | 0,235 |
| RAD18 | Hs.375684 | 1,006 | 0,84 | 0,984 | 0,527 | 0,984 | 0,583 |
| RAD21 | Hs.81848 | 1,059 | 0,157 | 0,96 | 0,239 | 1,047 | 0,112 |
| RAD50 | Hs.633509 | 1,006 | 0,702 | 1,03 | 0,221 | 0,979 | 0,154 |
| RAD51 | Hs.631709 | 1,029 | 0,577 | 0,998 | 0,962 | 1,017 | 0,736 |
| RAD51B | Hs.172587 | 1,032 | 0,531 | 1,036 | 0,489 | 1,033 | 0,521 |
| RAD9A | Hs.655354 | 0,838 | 0,256 | 0,795 | 0,172 | 0,959 | 0,763 |
| RBBP8 | Hs.546282 | 0,977 | 0,644 | 0,999 | 0,988 | 0,948 | 0,377 |
| REV1 | Hs.443077 | 1,035 | 0,329 | 1,033 | 0,392 | 1,034 | 0,227 |
| RNF168 | Hs.250648 | 0,933 | 0,589 | 0,931 | 0,566 | 0,98 | 0,869 |
| RNF8 | Hs.485278 | 1,019 | 0,757 | 1,012 | 0,804 | 1,044 | 0,559 |
| RPA1 | Hs.461925 | 1,052 | 0,39 | 1,029 | 0,232 | 1,051 | 0,083 |
| SIRT1 | Hs.369779 | 0,858 | 0,046 | 0,836 | 0,018 | 0,842 | 0,031 |
| SMC1A | Hs.211602 | 0,96 | 0,609 | 0,928 | 0,392 | 0,945 | 0,526 |
| SUMO1 | Hs.81424 | 0,987 | 0,671 | 0,989 | 0,711 | 0,924 | 0,178 |
| TOPBP1 | Hs.593379 | 1,021 | 0,649 | 0,989 | 0,656 | 0,988 | 0,709 |
| TP53 | Hs.437460 | 0,932 | 0,322 | 0,88 | 0,141 | 0,956 | 0,56 |
| TP53BP1 | Hs.440968 | 1,023 | 0,627 | 1,063 | 0,226 | 1,028 | 0,563 |
| TP73 | Hs.192132 | 0,991 | 0,964 | 0,931 | 0,741 | 0,99 | 0,963 |
| UNG | Hs.191334 | 1,036 | 0,48 | 1,047 | 0,388 | 0,955 | 0,436 |
| XPA | Hs.654364 | 1,061 | 0,645 | 1,195 | 0,242 | 1,017 | 0,893 |
| XPC | Hs.475538 | 1,083 | 0,394 | 1,128 | 0,075 | 1,061 | 0,598 |
| XRCC1 | Hs.98493 | 1,027 | 0,257 | 1,005 | 0,868 | 1,026 | 0,216 |
| XRCC2 | Hs.647093 | 1,101 | 0,167 | 1,211 | 0,040 | 1,246 | 0,045 |
| XRCC3 | Hs.592325 | 0,878 | 0,651 | 0,748 | 0,388 | 0,967 | 0,917 |
| XRCC6 | Hs.292493 | 1,12 | 0,082 | 1,087 | 0,163 | 1,061 | 0,439 |
